# Supplementary material for: Coordinated repression of totipotency-associated gene loci by histone methyltransferase EHMT2 via LINE1 regulatory elements
Source: EMBO Rep. 2025 Dec 9;27(3):654–76. doi: 10.1038/s44319-025-00657-5 (PMC12894760; doi:10.1038/s44319-025-00657-5)
Supplement: Supplementary file 1 — Appendix [file 44319_2025_657_MOESM1_ESM.pdf]

Appendix

Table of Contents

Appendix Figure S1 – page 2

Appendix Figure S2 – page 4

Appendix Figure S3 – page 6

Appendix Figure S4 – page 8

Appendix Figure S5 – page 9

Appendix Figure S6 – page 10

Appendix Figure S7 – page 11

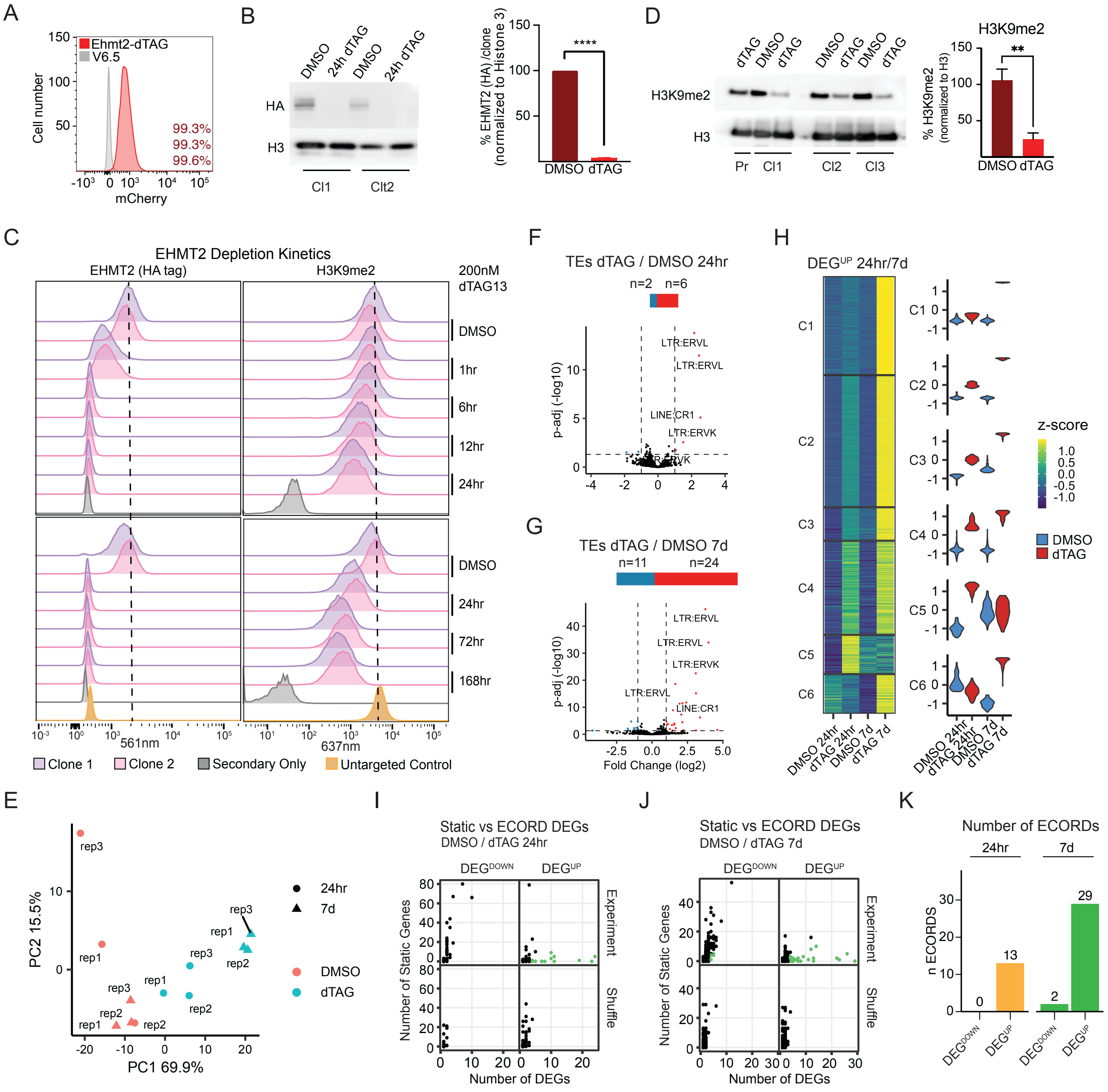

Appendix Figure S1. Acute EHMT2 depletion reveals distinct categories of target genes. Related to Figure 1.

A) Flow cytometric analysis of mCherry expression in n=3 independent EHMT2-dTAG mESC lines. **B)** Western blot of EHMT2 (HA) levels after 24h of dTAG treatment. ****p<0.0001; two-tailed, paired t-test. Error bars represent the mean with SD. **C)** Flow cytometry time course of EHMT2 (HA) and H3K9me2 levels during continuous dTAG treatment of EHMT2-dTAG mESCs. **D)** Western blot of H3K9me2 levels after 24h of dTAG treatment. **p<0.01; two-tailed, unpaired t-test. Error bars represent the mean with SD. **E)** PCA of RNA-seq samples. **F-G)** Volcano plots

showing numbers of differentially expressed (adj. P-value <0.05 & absolute log2 fold change ≥1) repetitive elements (TEs) in response to 24h and 7d continuous dTAG treatment, respectively. **H)** K-medoids clustering of all DEGs after 24h or 7d dTAG treatment. Genes are z-scored by row. **I-J)** Number of static genes in ECORDs at 24h and 7d, respectively. Green indicates ECORDs with ≥4 DEGs. **K)** The number of ECORDs detected by RNA-seq after 24h and 7d of dTAG treatment.

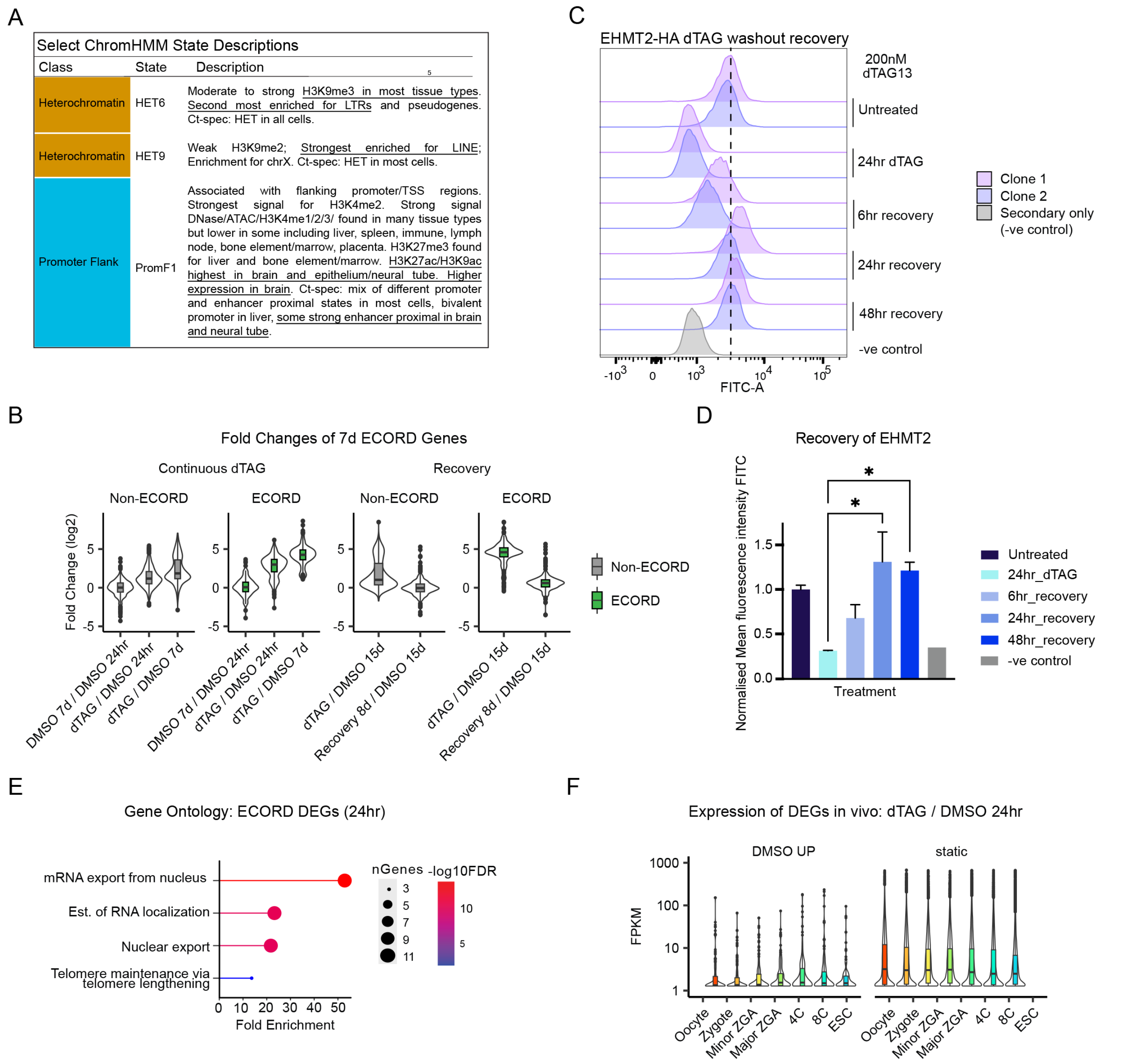

Appendix Figure S2. Genes in ECORDs have distinct properties and are enriched for genes involved in Zygotic Genome Activation (ZGA). Related to Figure 2.

A) The descriptions of the ChromHMM terms shown in Fig.2A. **B)** Fold-changes of 7d dTAG Up DEGs (Non-ECORD and ECORD) after 15d continuous dTAG treatment (left) and after 7d continuous dTAG treatment followed by 8d of dTAG washout ("Recovery", right), as measured by RNA-seq. **C)** Flow cytometry time course showing EHMT2 (HA) recovery after dTAG washout. A sample treated with secondary antibody only was used as a negative (-ve) control. **D)** Quantification of the mean fluorescence intensity measurements from C. *$p<0.05$; One-way ANOVA. Error bars represent the mean with SD. **E)** Gene ontology (GO) terms of ECORD DEGs

after 24h of dTAG treatment. **F)** Expression of 24h DMSO Up DEGs and static genes in an *in vivo* time course of embryonic development (Wu et al., 2016, and Hu et al., 2020). Only genes with an FPKM ≥10 in at least one sample type were included.

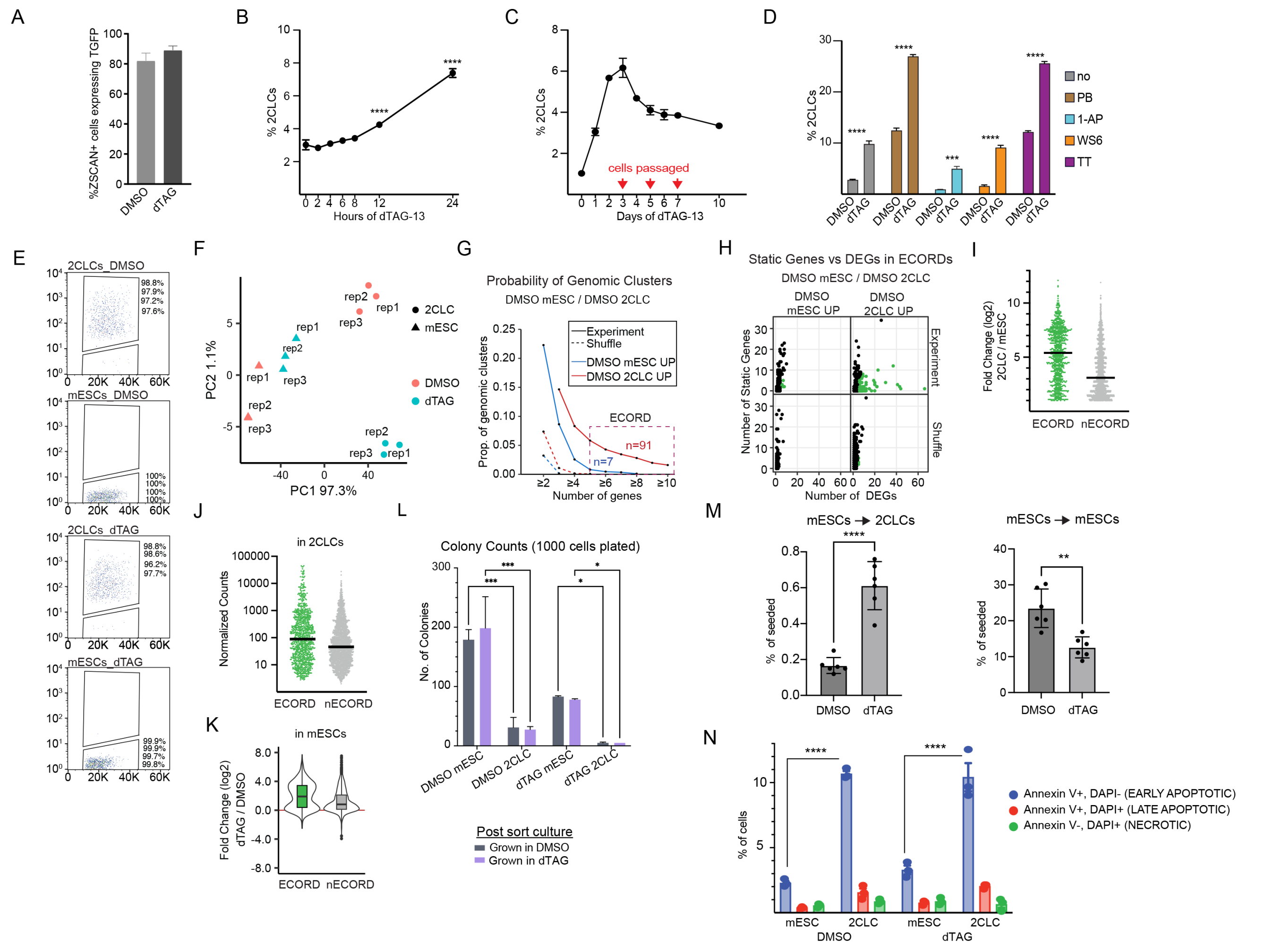

Appendix Figure S3. EHMT2 acts as a gatekeeper for entry into the 2-cell-like state. Related to Figure 3.

A) Quantification of the percentage of ZSCAN4-positive cells that express GFP (TGFP) in DMSO and dTAG conditions. Error bars represent the mean with SD. **B)** Percentage of 2CLCs in mESC cultures after indicated hours of dTAG-13 treatment compared to DMSO-treated (0h) cells. ****p<0.0001 with one-way ANOVA. Shown is the mean with SD (n=3 biological replicates). **C)** Percentage of 2CLCs after indicated days of dTAG-13 treatment. Shown is the mean with SD. Red arrows indicate when mESC cultures were passaged. **D)** Percentage of 2CLCs after 24h of mESC culture in the presence of the indicated chemicals. Shown is the mean with SD (n=3 biological replicates). Nothing added, no; Pladienolide B, PB (spliceosome inhibitor); 1-azakenpaullone, 1-AP (GSK3 inhibitor); WS6 (IkB kinase and EPB1 inhibitor); TTNPB, TT (RAR agonist). ****p<0.0001; one-Way Anova. **E)** FACS analysis indicating purity of 2CLC and mESC populations used for RNA-seq analysis. **F)** PCA of RNA-seq samples. **G)** Probability curves showing the fraction of gene clusters with ≥n DEGs and < 50% static genes that do not break

TAD boundaries. For RNA-seq experiments with sorted cells, “ECORDs” were defined as clusters with n ≥5 DEGs and <0.5 proportion of static genes. **H)** Number of static genes in ECORDs at 24h and 7d, respectively. Green indicates ECORDs with ≥5 DEGs. **I)** Fold change of ECORD and Non-ECORD Up DEGs (DMSO 2CLC > mESC). **J)** Normalized expression of ECORD and Non-ECORD Up DEGS (DMSO 2CLC > mESC) in DMSO 2CLC **K)** Fold changes of ECORD and Non-ECORD Up DEGs (mESC dTAG/DMSO) in mESC dTAG (mESC dTAG / DMSO). **L)** Quantification of Alkaline phosphatase-positive colonies after 6 days of culture of sorted populations in different media conditions. *p<0.05, ***p<0.001; two-way ANOVA. Error bars represent the mean with SD. **M)** Quantification of cell state conversions of sorted populations in different media conditions after 24 hours. **p<0.01, ****p<0.0001 with unpaired t-test. **N)** Quantification of indicated cell populations defined after Annexin V/DAPI staining of 2CLCs and mESCs. ****p<0.0001; two-way ANOVA. Error bars represent the mean with SD.

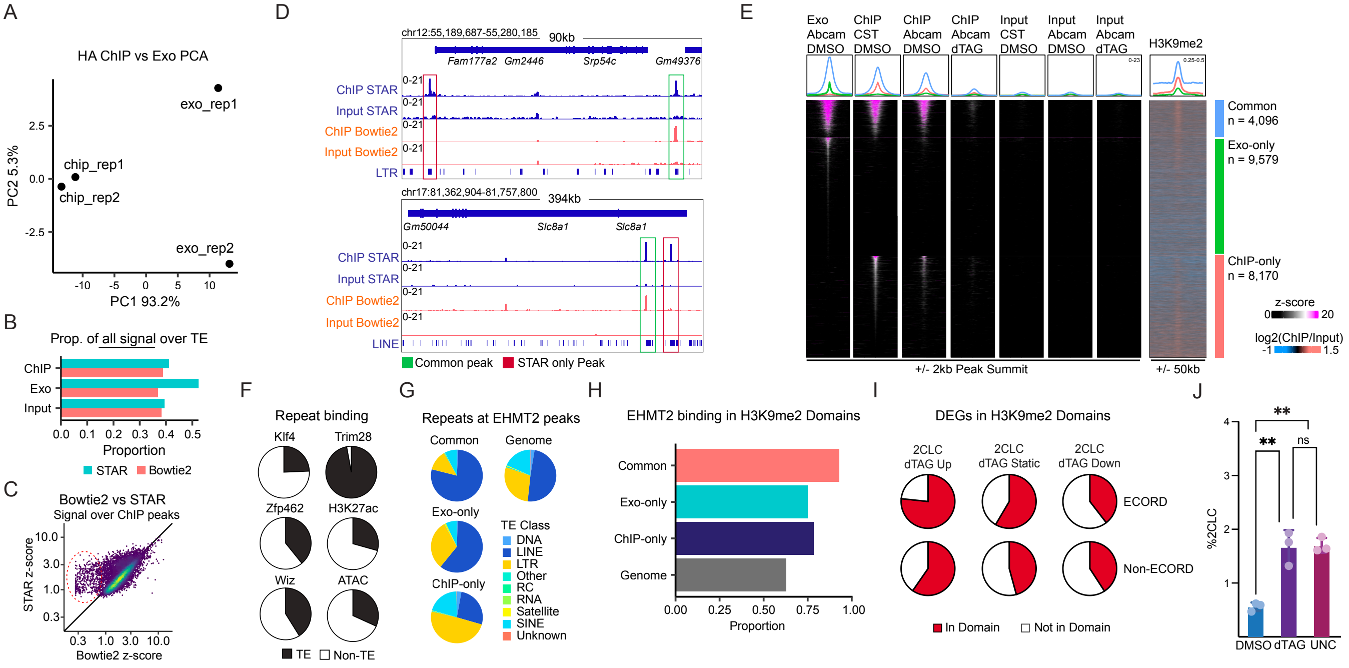

Appendix Figure S4. H3K9me2 domains repress ECORD expression. Related to Figure 4. **A)** PCA of ChIP-seq and ChIP-Exo samples. **B)** Comparison of Bowtie2 and modified STAR multi-alignment (see Methods) showing the proportion of signal over repeats. **C)** Correlation of EHMT2 ChIP-seq signal after aligning with Bowtie2 or STARmulti (see Methods). Most peaks are unaffected, but a subset of *de novo* peaks can be identified with STARmulti (dashed circle). **D)** Examples of peaks only identified using STARmulti (red box) and peaks unaffected by the aligner used (green box). **E)** EHMT2 and H3K9me2 signal around summits of ChIP-Exo and ChIP-seq peaks (observed in indicated culture conditions and using indicated HA antibodies. For common peaks, the mid-point of the summit was used. **F)** Proportion of peaks that overlap repetitive elements. Proportion was calculated at the base pair level. **G)** Classes of repetitive elements (TEs) overlapping with distinct categories of EHMT2 peaks. **H)** Proportion of EHMT2 ChIP-seq and ChIP-Exo peaks that overlap an H3K9me2 domain. **I)** Proportion of RNA-seq k-meds genes that overlap an H3K9me2 domain, split by whether they fall into an ECORD called in DMSO 2CLCs. **J)** Percentage of 2CLCs in mESC cultures after 24h of dTAG-13 or UNC0638 treatment compared to DMSO-treated. **p<0.01; one-way ANOVA. Error bars represent the mean with SD.

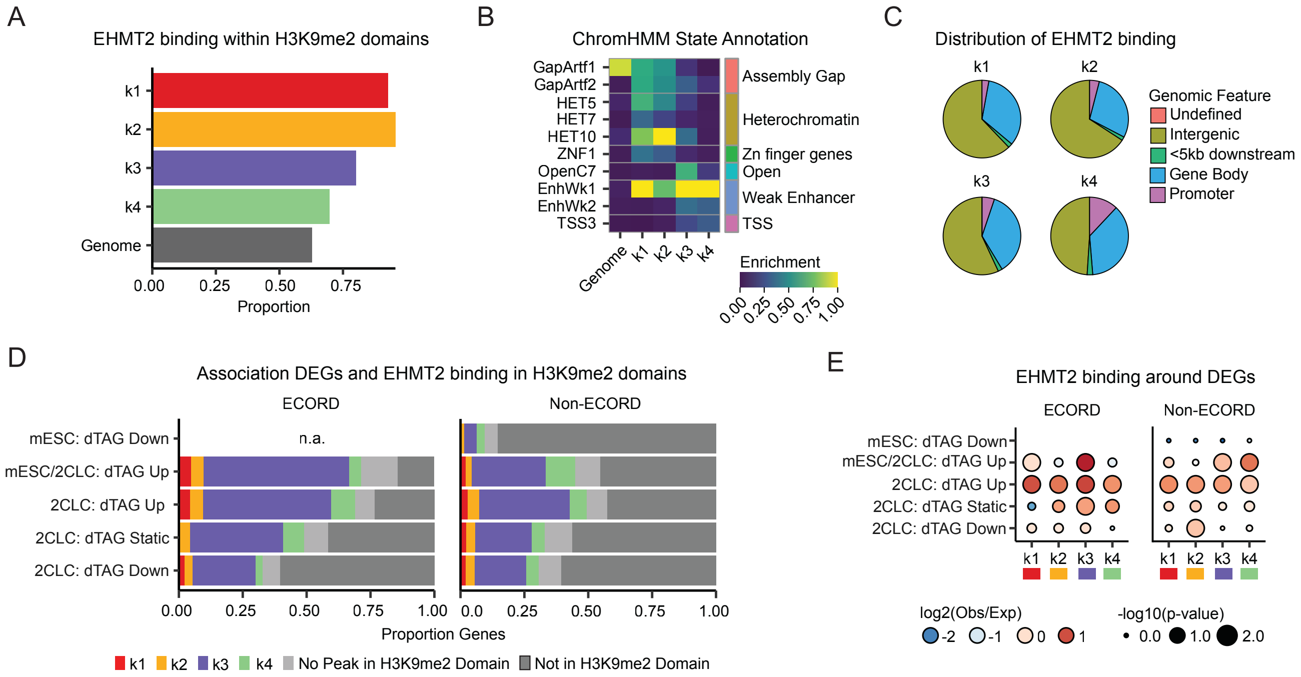

Appendix Figure S5. EHMT2 binding suggests locus-specific modes of gene regulation. Related to Figure 5.

A) Proportion of peaks in EHMT2 k-means clusters (k1 to k4) that overlap H3K9me2 domains. **B)** ChromHMM annotations of EHMT2 peaks using a 100-state model (Vu and Ernst, 2023). **C)** Genomic distribution of EHMT2 peaks in the different k-means clusters. **D)** Enrichment of EHMT2 k-means clusters with DEGs. DEGs were assigned to the nearest EHMT2 peak in the same H3K9me2 domain. The p-value was calculated using a bootstrap approach by shuffling gene categories 1,000 times. **E)** Association between RNA-seq DEGs, k-meds clusters, ECORDs, and EHMT2 binding. Genes were assigned to the nearest EHMT2 peak in the same H3K9me2 domain.

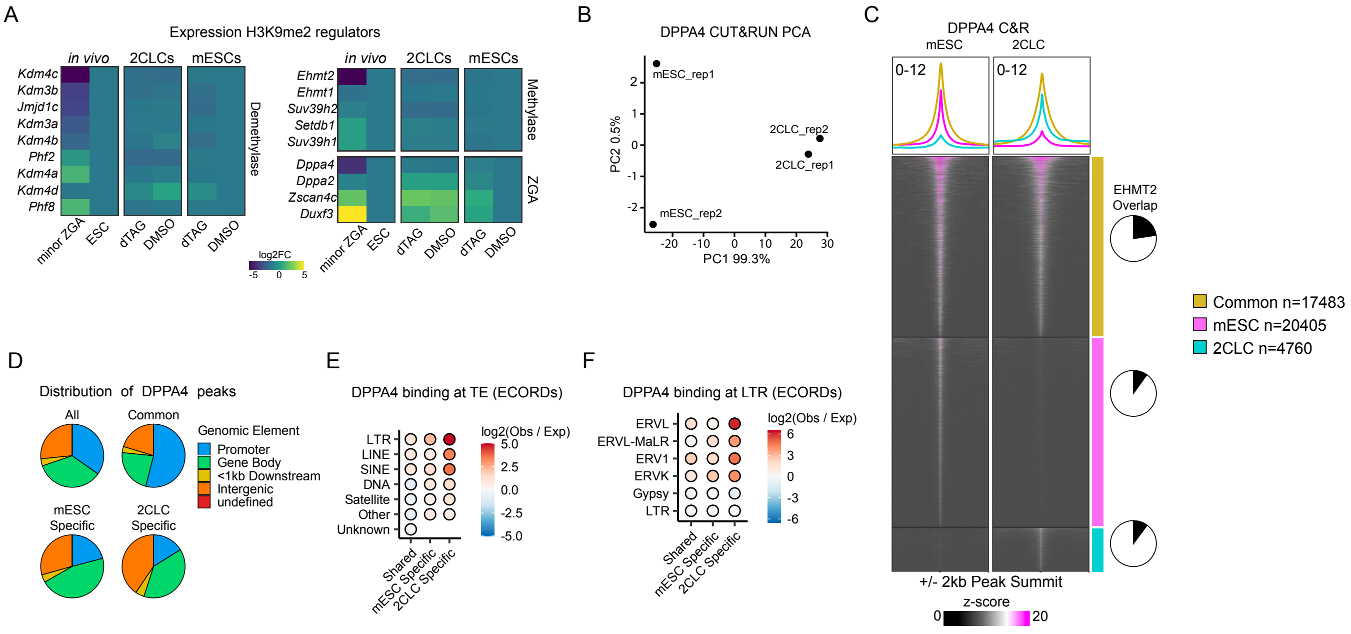

Appendix Figure S6. EHMT2 antagonizes DPPA2/4 function at ECORDs. Related to Figure 6.

A) Expression of H3K9me2 regulators in sorted 2CLCs and mESCs compared to mouse embryos at the minor ZGA stage and mouse ESCs from Hu et al., 2020. **B)** PCA of DPPA4 CUT&RUN samples. **C)** DPPA4 C&R signal (+/-2kb of peak summits) around all DPPA4 peaks, organized by their cell type association. For common peaks, the mid-point of the summits was used. Pie diagrams show overlap with EHMT2 binding based on a union set of EHMT2 ChIP-seq and ChIP-exo peaks. **D)** Genomic distribution of DPPA4 peaks. **E)** Observed vs expected ratio (log2) of DPPA4 peaks over different classes of repetitive elements (TEs). **EF)** Observed vs expected ratio (log2) of DPPA4 peaks over different families of LTRs. For this analysis, only peaks that overlapped an LTR were used. The expected ratio was calculated for D and E by shuffling Dppa4 peaks over the genome 100 times.

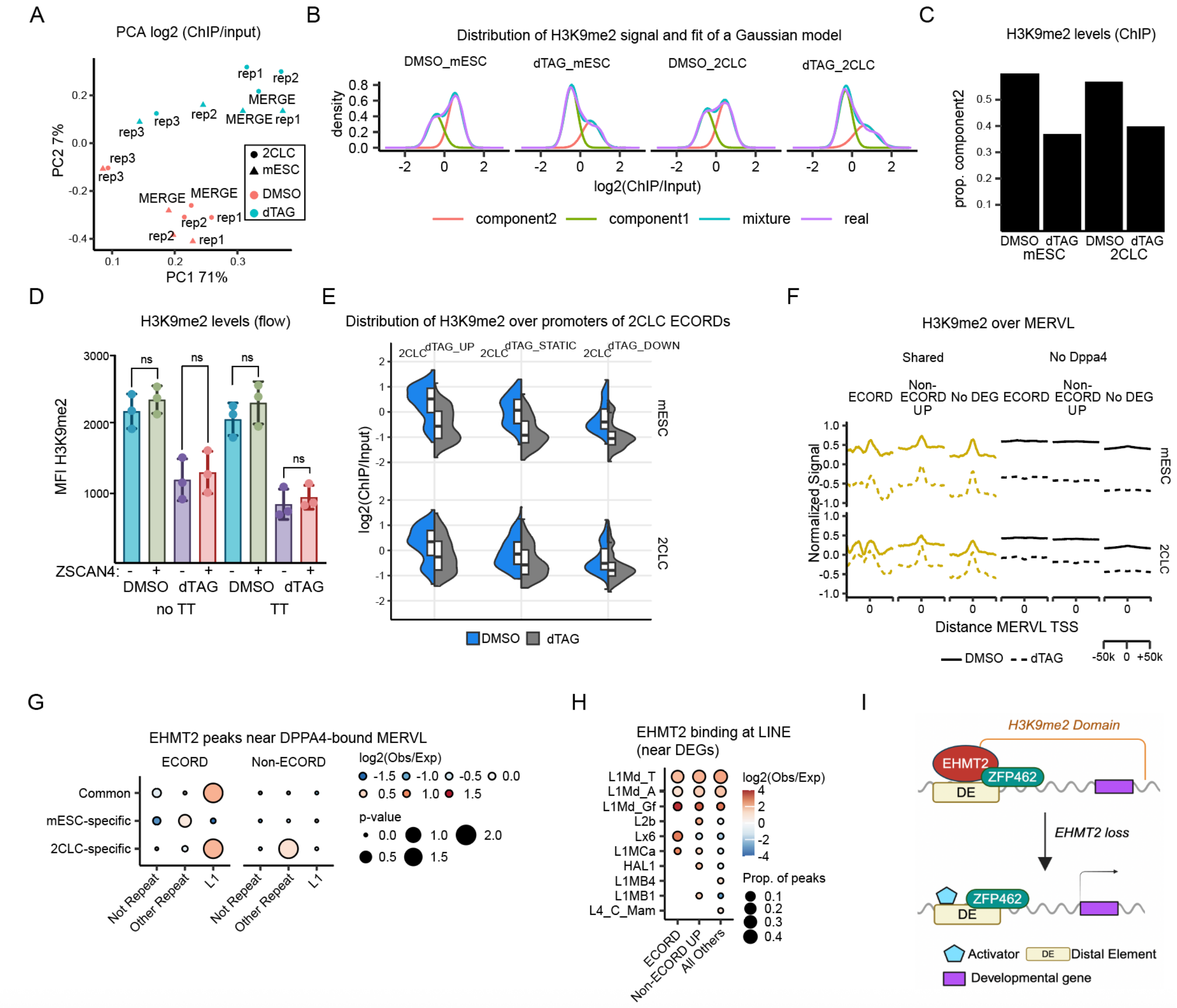

Appendix Figure S7: Evidence for a regulatory role of EHMT2-bound L1s for ECORD expression. Related to Figure 7

A) PCA of H3K9me2 ULI-NChIP samples based on ChIP/Input signal. **B)** Gaussian mixture modeling of H3K9me2 signal (ChIP/Input) using a two-component model. "real" represents the actual data and "mixture" is the model fit/prediction that is the sum of component 1 and component 2. **C)** Proportion of the total mixture curve from figure S7B represented by component 2, representing the (H3K9me2) signal component. **D)** Quantification of H3K9me2 levels in mESCs (ZSCAN4-) and 2CLCs (ZSCAN4+) derived in either presence or absence of the RAR agonist TT by flow cytometry after treatment with DMSO/dTAG for 48h. n.s: not significant ($p>0.05$) with one-way ANOVA. Error bars represent the mean with SD. **E)** H3K9me2

signal in mESCs and 2CLCs (DMSO, dTAG) over gene TSSs for indicated gene groups defined by RNA-seq analysis of sorted 2CLCs (dTAG vs DMSO) (see Figure 3I). **F)** H3K9me2 signal over MERVLs bound by DPPA4 in both 2CLCs and mESCs (yellow) or not bound at all (black). The H3K9me2 signal in panels E and F represents the log2(ChIP/Input) signal, which is additionally normalized using a scaling factor estimated by mixture modeling (see Methods). **G)** Enrichment of EHMT2 peaks around Dppa4 MERVL at ECORDs and Non-ECORD UP DEGs. Dppa4 peaks were assigned to the nearest EHMT2 peak in the same H3K9me2 domain. The p-value was calculated using a bootstrap approach by shuffling Dppa4 peaks over the genome 1,000 times. **H)** Observed vs expected ratio (log2) of LINE repeat genes that EHMT2 binds to, split by the type of DEG they are associated with. To associate peaks and DEGs, each DEG was assigned to the nearest EHMT2 peak in the same H3K9me2 domain (see methods). **I)** Model of EHMT2 regulation of non-ECORD genes.
