# Supplementary material for: Coordinated repression of totipotency-associated gene loci by histone methyltransferase EHMT2 via LINE1 regulatory elements
Source: EMBO Rep. 2025 Dec 9;27(3):654–76. doi: 10.1038/s44319-025-00657-5 (PMC12894760; doi:10.1038/s44319-025-00657-5)
Supplement: Supplementary file 10 — Source data Fig. 3 [file 44319_2025_657_MOESM10_ESM.zip › Figure 3/3C/README.docx]

FCS files of flow cytometric analysis of MERVL-GFP mESC lines #7, #9, and #11 for expression of EpCAM and SSEA1 on GFP- (mESCs) and GFP+ (2CLCs) in presence of DMSO or dTAG. Gating was used to exclude feeder cells (high FSC and SSC values), dead cells (DAPI+) and duplets.
