# Supplementary material for: Coordinated repression of totipotency-associated gene loci by histone methyltransferase EHMT2 via LINE1 regulatory elements
Source: EMBO Rep. 2025 Dec 9;27(3):654–76. doi: 10.1038/s44319-025-00657-5 (PMC12894760; doi:10.1038/s44319-025-00657-5)
Supplement: Supplementary file 10 — Source data Fig. 3 [file 44319_2025_657_MOESM10_ESM.zip › Figure 3/3D/README.docx]

README

MERVL-GFP mESC clone 7 was used in biological triplicate (three separate cultures).

24h = analysis after 24 hours of culture in dTAG

no = analysis after 24 hours of culture in DMSO

Parental EHMT2-dTAG mESCs were used to determine background GFP levels.

For panel 3D, samples 24h_1 and no_1 were used.
