# Supplementary figures and images for: Coordinated repression of totipotency-associated gene loci by histone methyltransferase EHMT2 via LINE1 regulatory elements
Source: EMBO Rep. 2025 Dec 9;27(3):654–76. doi: 10.1038/s44319-025-00657-5 (PMC12894760; doi:10.1038/s44319-025-00657-5)

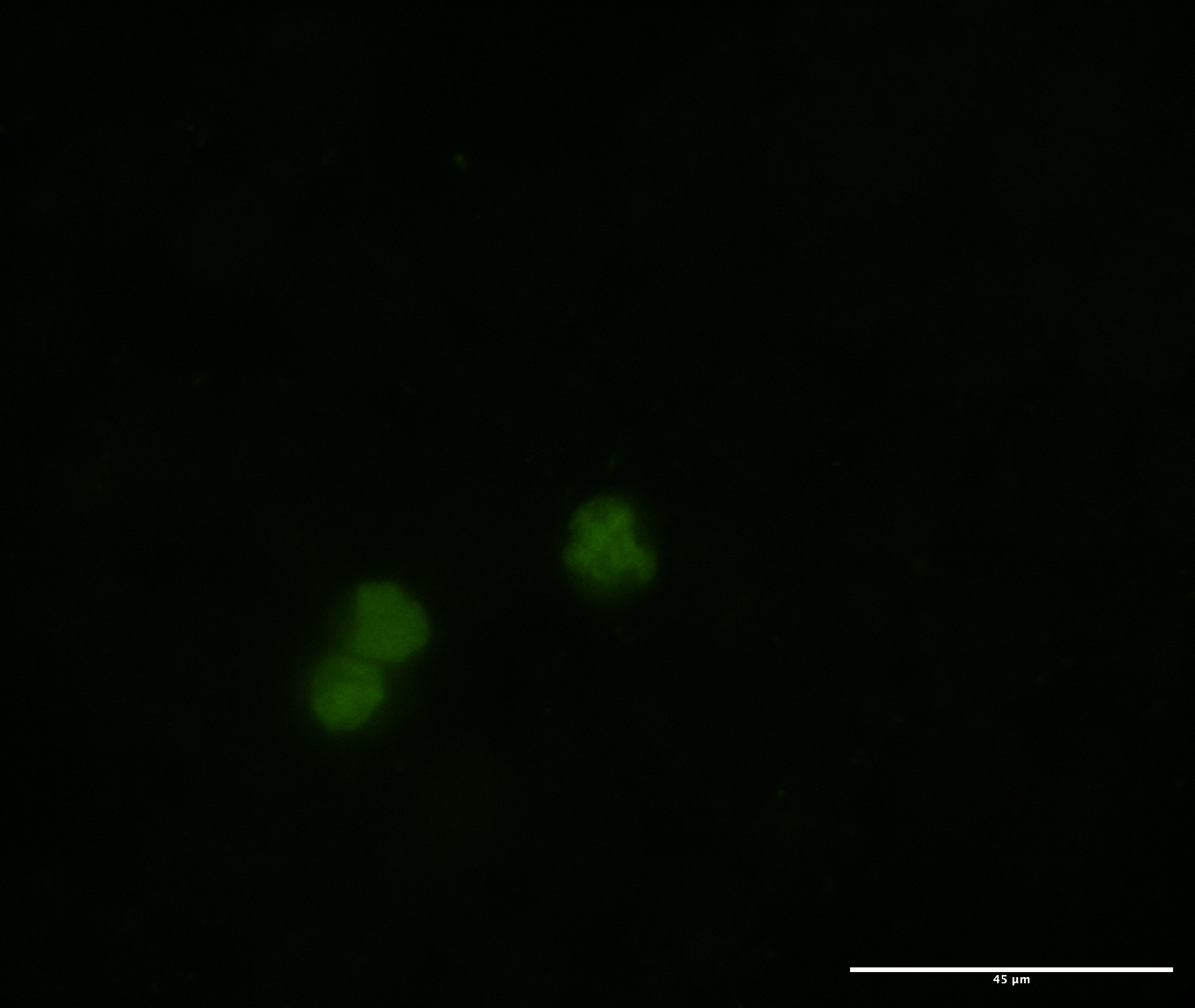

Supplement: Supplementary file 10 — Source data Fig. 3 [file 44319_2025_657_MOESM10_ESM.zip › Figure 3/3B/TurboGFP.jpg]

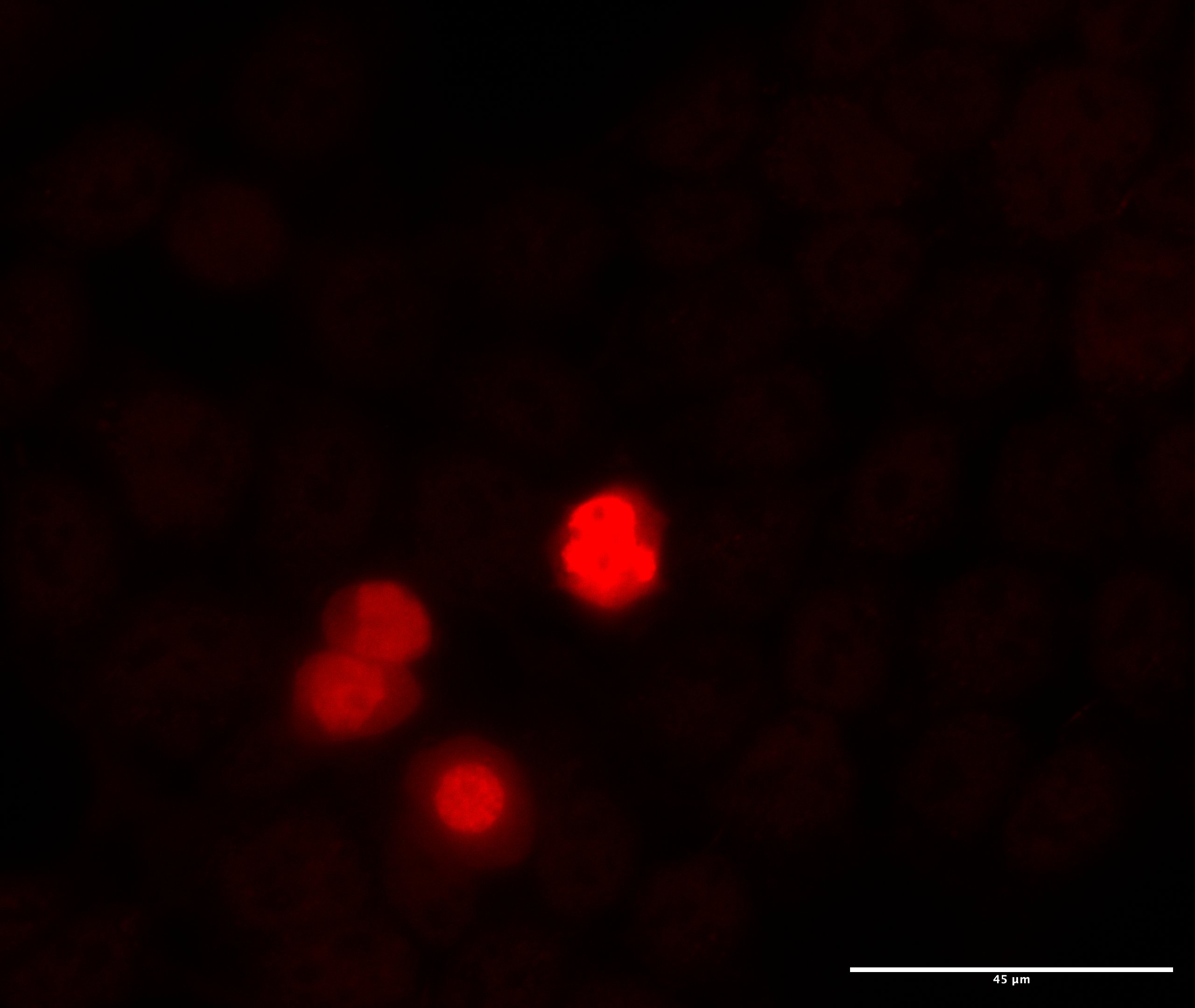

Supplement: Supplementary file 10 — Source data Fig. 3 [file 44319_2025_657_MOESM10_ESM.zip › Figure 3/3B/ZSCAN4.jpg]

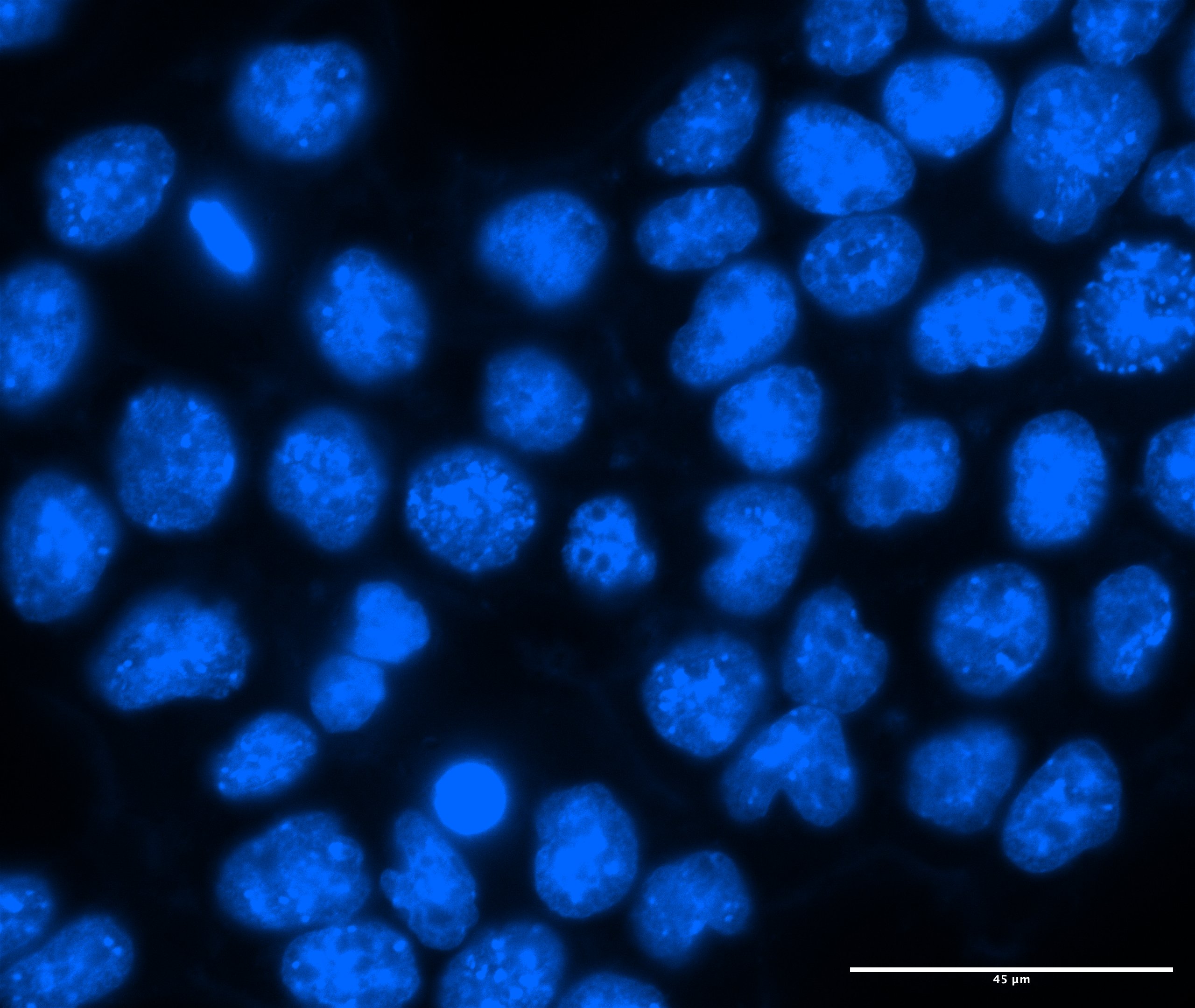

Supplement: Supplementary file 10 — Source data Fig. 3 [file 44319_2025_657_MOESM10_ESM.zip › Figure 3/3B/DAPI.jpg]
