# Supplementary material for: Coordinated repression of totipotency-associated gene loci by histone methyltransferase EHMT2 via LINE1 regulatory elements
Source: EMBO Rep. 2025 Dec 9;27(3):654–76. doi: 10.1038/s44319-025-00657-5 (PMC12894760; doi:10.1038/s44319-025-00657-5)
Supplement: Supplementary file 12 — Source data Fig. 5 [file 44319_2025_657_MOESM12_ESM.zip › Figure 5/5E/README.docx]

Table listing gene categories and RNA-seq fold-changes, as used in Fig.5E.

gene.id = common gene name

Cell = higher expressed in mESC (“ESC”), 2CLCs (“2CLC”), or not differential, based on RNA-seq analysis.

Category – Part of ECORD and upregulated in dTAG (“ECORD”); not part of ECORD and upregulated in dTAG (“Non-ECORD UP”); upregulated in DMSO (“DMSO UP”).

Log2FC_UNC:DMSO_WT - average log2FC difference in RNA levels comparing J1 wildtype mESCs grown in UNC0638 and DMSO)

Log2FC_UNC:DMSO_DPPA4KO - average log2FC difference in RNA levels comparing DPPA4KO mESCs grown in UNC0638 and DMSO)

See Table S2 (sheet “7dDEGs_UNC_DPPA4WTvsKO”) for additional information on this dataset.
